# Value analysis of preoperative NRS2002 and GLIM screening in predicting postoperative complications in patients with gastrointestinal malignancies

**DOI:** 10.3389/fnut.2025.1641539

**Published:** 2025-10-17

**Authors:** Dongping Xu, Huaying Yan, Jingjing Wang, Ping Zheng

**Affiliations:** ^1^Department of Nutrition, The Third Central Hospital of Tianjin, Tianjin, China; ^2^Tianjin Key Laboratory of Extracorporeal Life Support for Critical Diseases, Tianjin, China; ^3^Artificial Cell Engineering Technology Research Center, Tianjin, China; ^4^Tianjin Institute of Hepatobiliary Disease, Tianjin, China

**Keywords:** Nutritional Risk Screening 2002 (NRS2002), Global Leadership Initiative on Malnutrition (GLIM), gastrointestinal malignancies, postoperative complications, prognostic value, nutritional assessment

## Abstract

**Objective:**

To investigate and compare the predictive efficacy of the Nutritional Risk Screening 2002 (NRS2002) and the Global Leadership Initiative on Malnutrition (GLIM) criteria for postoperative complications in patients with gastrointestinal malignancies.

**Methods:**

This prospective cohort study enrolled patients with gastrointestinal malignancies scheduled for surgical resection at our institution from December 2019 to December 2024. Nutritional risk and status were assessed using NRS2002 and GLIM criteria within 24 h of admission. Data on postoperative complications, hospitalization expenses, length of hospital stay, and unplanned 30-day and 60-day readmissions were meticulously collected and analyzed using logistic regression and ROC curve analysis.

**Results:**

A total of 471 patients were included. Nutritional risk (NRS2002 ≥ 3) was identified in 45.01% (*n* = 212) of patients. According to GLIM criteria, 43.10% (*n* = 203) were diagnosed with malnutrition. Multivariable logistic regression analysis revealed that nutritional risk (adjusted OR 7.58, 95%CI: 4.75–12.05, *p* < 0.001), GLIM-defined malnutrition (adjusted OR 5.62, 95%CI: 3.59–8.76, *p* < 0.001), moderate malnutrition (adjusted OR 4.78, 95%CI: 2.78–8.17, *p* < 0.001), and severe malnutrition (adjusted OR 6.71, 95%CI: 3.82–11.78, *p* < 0.001) were independent risk factors for postoperative complications. The Area Under the Curve (AUC) for NRS2002 in predicting complications was 0.735, which was significantly greater than the AUC for GLIM diagnosis (0.706; *p* = 0.003). No significant difference in AUC was observed between NRS2002 and GLIM severity grading (0.712; *p* = 0.215). Neither NRS2002 nor GLIM assessments were significantly associated with unplanned readmissions in adjusted models.

**Conclusion:**

Both NRS2002-defined nutritional risk and GLIM-defined malnutrition are significant independent predictors of postoperative complications in patients with gastrointestinal malignancies. The NRS2002 demonstrated slightly superior predictive ability for postoperative complications compared to the dichotomous GLIM diagnosis. These findings support the routine use of both tools for preoperative risk stratification to guide targeted nutritional interventions.

## 1 Introduction

Malignant tumors represent a growing public health concern globally, with their incidence and mortality rates exhibiting a continuous upward trajectory in many regions (1). Gastrointestinal (GI) cancers, particularly colorectal and gastric cancers, rank among the most common malignancies and are significant contributors to cancer-related mortality (2). Malnutrition is a prevalent and serious comorbidity in cancer patients, with reported incidences particularly high in individuals with GI malignancies, affecting up to 80% of this population (3, 4). It is estimated that approximately 20% of cancer-related deaths are directly attributable to malnutrition rather than the tumor burden itself (5). Surgical resection remains the cornerstone of curative treatment for most localized GI malignancies. However, the physiological stress of surgery can rapidly deplete the body’s nutritional reserves, impairing functional recovery and wound healing processes. Consequently, malnourished patients are at an elevated risk for postoperative complications, prolonged hospital stays, increased healthcare costs, and compromised long-term outcomes (4, 6).

In clinical settings, malnutrition is recognized as a complex state of nutritional deficiency that adversely impacts patient outcomes (7, 8). The diagnostic pathway for malnutrition in many healthcare systems, including China, typically involves a hierarchical approach: nutritional screening, nutritional assessment, and comprehensive evaluation (9). The Nutritional Risk Screening 2002 (NRS2002) is a widely adopted, evidence-based tool for identifying patients at nutritional risk who may benefit from nutritional support (10, 11). Its integration into routine hospital admission procedures has become standard practice, often guiding indications for nutritional therapy. However, the subjective component of disease severity scoring in NRS2002 has been a point of discussion.

Concurrently, the Global Leadership Initiative on Malnutrition (GLIM) introduced a set of consensus-based, universally applicable criteria for diagnosing and grading malnutrition in adults in 2018 (12, 13). The GLIM framework aims to standardize malnutrition diagnosis worldwide, facilitating better research comparability and clinical management. Since its introduction, several studies have validated GLIM criteria for predicting survival and other outcomes in various cancer populations, including head and neck (14), lung (15), and GI cancers (16, 17). Nevertheless, the relationship between GLIM-defined malnutrition and specific treatment outcomes, such as postoperative complications and unplanned readmissions following major surgery for GI malignancies, warrants more extensive investigation.

This study aimed to evaluate and compare the predictive value of NRS2002 (utilizing the 2022 expert consensus for disease severity scoring) and GLIM criteria (including malnutrition severity) for postoperative complications and unplanned readmissions in a cohort of patients undergoing surgical resection for GI malignancies. The findings are intended to provide evidence-based insights for the clinical application of these nutritional assessment tools in the perioperative management of this vulnerable patient group.

## 2 Participants and methods

### 2.1 Study population and design

This prospective cohort study was conducted at our hospital, a tertiary care center. Patients with a new diagnosis of gastrointestinal malignant tumors scheduled for elective radical surgery between December 2019 and December 2024 were consecutively enrolled. Inclusion criteria were: (1) age ≥18 years; (2) histopathologically confirmed primary GI malignancy (e.g., esophageal, gastric, colorectal cancer); (3) planned curative-intent surgical resection; (4) no prior anti-tumor treatments such as surgery, radiotherapy, chemotherapy, or immunotherapy for the current malignancy; and (5) provision of written informed consent to participate in the study. Exclusion criteria included: (1) presence of other concurrent systemic malignant tumors; (2) emergency surgery; (3) incomplete critical clinical or nutritional data for baseline assessment.

The study protocol was approved by the Institutional Review Board and Ethics Committee of The Third Central Hospital of Tianjin (Approval No. SZX-IRB-SOP-016(F)-002–01). All procedures were performed in accordance with the Declaration of Helsinki and its later amendments.

### 2.2 Data collection

Comprehensive baseline data were collected within 24 h of admission. This included: demographic information (age, gender, education level), lifestyle factors (smoking, alcohol consumption), comorbidities (e.g., diabetes, hypertension, cardiovascular disease, Charlson Comorbidity Index), anthropometric measurements [height, weight, Body Mass Index (BMI)], NRS2002 score, GLIM malnutrition assessment, Karnofsky Performance Status (KPS) score, and preoperative laboratory parameters (e.g., albumin, prealbumin, hemoglobin, total lymphocyte count, C-reactive protein).

Perioperative data included: primary tumor site, surgical procedure details (type, approach, e.g., open vs. minimally invasive, duration), American Society of Anesthesiologists (ASA) physical status classification, pathological diagnosis (tumor type, grade), final Tumor Node Metastasis (TNM) staging (AJCC 8th edition), administration of perioperative nutritional support, occurrence and nature of postoperative complications, intensive care unit (ICU) admission, total length of hospital stay, and hospitalization costs. The variable ‘administration of perioperative nutritional support’ was recorded dichotomously (yes/no) based on whether the patient received any form of specialized nutrition therapy (enteral or parenteral) during the perioperative period. Patients were followed up telephonically or during outpatient visits to ascertain unplanned readmission status at 30 and 60 days post-discharge.

**Figure 1:**
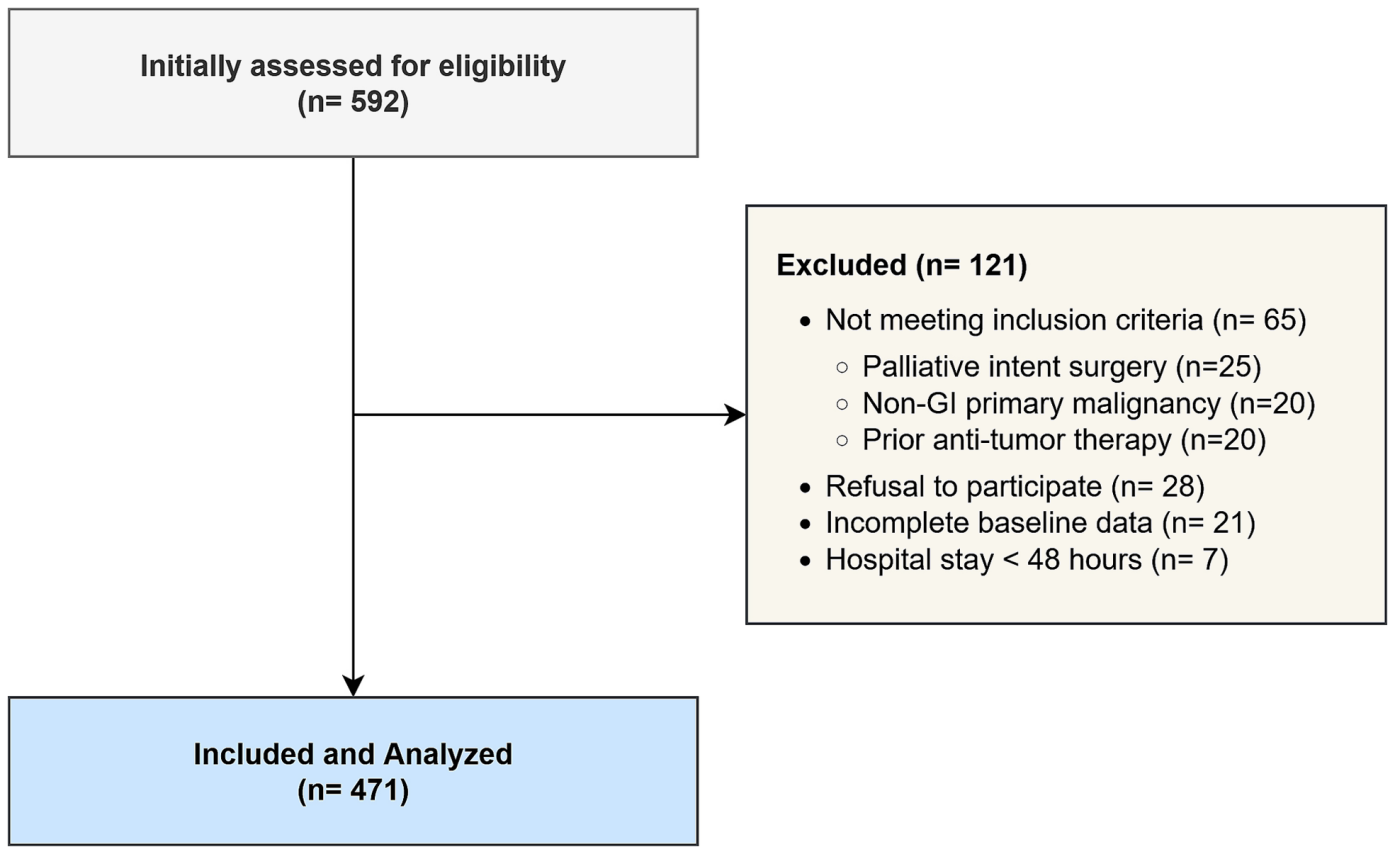
STROBE flow diagram of patient enrollment.

The patient selection process is illustrated in Figure 1. A total of 592 patients undergoing surgical resection for gastrointestinal malignancies between December 2019 and December 2024 were initially assessed for eligibility. Of these, 121 patients were excluded from the final analysis for the following reasons: not meeting inclusion criteria (*n* = 65), refusal to participate or provide informed consent (*n* = 28), and incomplete baseline data (*n* = 21). Additionally, 7 patients were eliminated post-enrollment due to a hospital stay of less than 48 h, as this short duration precluded the adequate assessment of postoperative complications. Consequently, 471 patients who met all criteria and completed the study protocol were included in the final analysis.

### 2.3 Nutritional screening and assessment

#### 2.3.1 Nutritional Risk Screening 2002 (NRS2002)

Nutritional risk was assessed using the NRS2002 tool. This involves an initial four-question screening. If any question is answered affirmatively, a final screening is performed, which scores impaired nutritional status (0–3 points) and disease severity (0–3 points). An age correction (+1 point) is added for patients ≥70 years. A total score ≥3 indicates nutritional risk, while a score <3 indicates no significant nutritional risk (10). The 2022 expert consensus guidelines were referenced for disease severity scoring (18). Specifically, disease severity was scored based on the consensus guidelines which categorize major abdominal surgery, such as that undertaken by our cohort, as a score of 2. The score was increased to 3 if there was evidence of severe systemic inflammation (e.g., sepsis, major trauma) or malignancy-related cachexia, allowing for a standardized application of the tool.

#### 2.3.2 Global Leadership Initiative on Malnutrition (GLIM) criteria

Malnutrition was diagnosed according to GLIM criteria (12), which require the presence of at least one phenotypic criterion and one etiologic criterion. Phenotypic criteria include: non-volitional weight loss (>5% within past 6 months, or >10% beyond 6 months), low BMI (<18.5 kg/m^2^ for Asians <70 years or <20.0 kg/m^2^ for Asians ≥70 years), and reduced muscle mass. Etiologic criteria include: reduced food intake or assimilation (≤50% of estimated energy requirements for >1 week, or any reduction for ≥2 weeks, or chronic GI conditions impacting absorption) and inflammation (acute disease/injury-related or chronic disease-related, evidenced by C-reactive protein >10 mg/L or clinical signs). Muscle mass was assessed based on the availability of clinical measurements. For approximately 35% of patients (*n* = 165) who had a recent abdominal CT scan for staging purposes, muscle mass was quantified by measuring the skeletal muscle index (SMI) at the third lumbar vertebra (L3), with cutoffs for low muscle mass set at <40.8 cm^2^/m^2^ for males and <34.8 cm^2^/m^2^ for females (8). For the remaining 65% of patients (*n* = 306), anthropometric proxies were used, specifically calf circumference (CC), with low muscle mass defined as CC <34 cm for males and <33 cm for females, according to Asian-specific recommendations validated for clinical use (16). Malnutrition severity was graded based on phenotypic criteria: moderate malnutrition (Stage I: weight loss 5–10% within 6 months, or mild-to-moderate deficit in muscle mass) and severe malnutrition (Stage II: weight loss >10% within 6 months, or severe deficit in muscle mass) (12).

### 2.4 Outcome measures

The primary outcome was the incidence of postoperative complications occurring during the index hospitalization, up to 30 days post-surgery. Complications were defined and graded according to the Clavien-Dindo classification system (19), and also categorized based on the “Expert Consensus on Postoperative Complications Registration for Gastrointestinal Tumor Surgery (2018 Edition)” (20). Major complications were defined as Clavien-Dindo grade III or higher. Secondary outcomes included length of hospital stay, total hospitalization costs, ICU admission rates, and unplanned readmission rates at 30 and 60 days post-discharge.

### 2.5 Statistical analysis

Statistical analyses were performed using R software (version 4.2.3, R Foundation for Statistical Computing, Vienna, Austria). Categorical variables were presented as frequencies and percentages (*n*, %), and continuous variables as mean ± standard deviation (SD) or median and interquartile range (IQR), based on data distribution (assessed by Shapiro–Wilk test). Comparisons between groups (e.g., complications vs. no complications) were made using Chi-square (*χ*^2^) test or Fisher’s exact test for categorical variables, and independent samples t-test or Mann–Whitney U test for continuous variables.

Univariable and multivariable logistic regression analyses were performed to identify independent predictors of postoperative complications and unplanned readmissions. Variables with *p* < 0.10 in univariable analysis, along with clinically relevant factors, were included in the multivariable models. Odds Ratios (ORs) with 95% Confidence Intervals (CIs) were calculated. Model adequacy was assessed using the Hosmer-Lemeshow goodness-of-fit test, and multicollinearity was checked using the Variance Inflation Factor (VIF), with a VIF >5 indicating potential collinearity. Overall model performance was summarized using Nagelkerke’s *R*^2^.

The predictive performance of NRS2002 score, GLIM malnutrition diagnosis, and GLIM severity grading for postoperative complications was evaluated using Receiver Operating Characteristic (ROC) curve analysis. The Area Under the Curve (AUC) with 95% CI was calculated for each model. AUCs were compared using DeLong’s test. A two-sided *p*-value <0.05 was considered statistically significant for all analyses. A subgroup analysis was conducted to assess the predictive performance of NRS2002 within different primary tumor sites (esophageal/gastric vs. colorectal).

### 2.6 Sample size calculation

The sample size was calculated based on the primary objective of identifying nutritional risk as a predictor of postoperative complications. Based on previous literature, the incidence of postoperative complications in non-malnourished GI cancer patients is approximately 20%. We aimed to detect at least a 15% increase in the complication rate (to 35%) in patients with nutritional risk. Using a two-sided significance level (*α*) of 0.05 and a statistical power (1−*β*) of 80%, the required sample size was estimated to be at least 199 patients per group. Considering a potential dropout rate of 10%, a total sample size of at least 440 patients was targeted. Our final cohort of 471 patients was therefore deemed sufficient for the planned analyses.

## 3 Results

### 3.1 Baseline characteristics of the study cohort

A total of 471 patients who underwent surgery for GI malignancies were included in the final analysis. The cohort comprised 319 (67.7%) males and 152 (32.3%) females. The median age of the cohort was 65.0 years (IQR: 58.0, 72.0 years). Based on NRS2002, 212 patients (45.01%) were identified as being at nutritional risk. According to GLIM criteria, 203 patients (43.10%) were diagnosed with malnutrition; of these, 95 (20.17% of total, 46.8% of malnourished) were classified as having moderate malnutrition (Stage I) and 85 (18.05% of total, 41.9% of malnourished) as having severe malnutrition (Stage II). The remaining 23 patients (4.88% of total, 11.3% of malnourished) met the GLIM criteria for malnutrition but could not be definitively graded into moderate/severe categories based on the combination of phenotypic criteria available; they were included in the overall ‘malnutrition’ group for dichotomous analysis but were excluded from the severity-graded analyses and ROC Model 3.

Patients were divided into two groups based on the occurrence of postoperative complications: a complication-free group (*n* = 317, 67.30%) and a complication group (*n* = 154, 32.70%). As shown in Table 1, significant differences were observed between the groups in terms of age, BMI, prevalence of comorbidities, ASA classification, KPS score, NRS2002-defined nutritional risk, GLIM-defined malnutrition, and severity of malnutrition (all *p* < 0.05). Specifically, patients in the complication group were older, had lower BMI, higher ASA scores, lower KPS scores, and were more likely to be at nutritional risk or have malnutrition. No significant intergroup differences were noted for gender, education level, smoking history, alcohol history, primary tumor site, surgical method, tumor differentiation, TNM stage, or receipt of perioperative nutritional support (all *p* > 0.05).

**Table 1:** Baseline clinical and nutritional characteristics of patients.

Parameter	Without complications (*n* = 317)	With complications (*n* = 154)	*p*-value
Age (years), median (IQR)	63.0 (56.0, 70.0)	68.0 (62.0, 75.0)	<0.001
Gender, *n* (%)			0.652
Male	217 (68.5)	102 (66.2)	
Female	100 (31.5)	52 (33.8)	
BMI (kg/m^2^), median (IQR)	22.5 (20.4, 24.8)	20.1 (18.2, 22.3)	<0.001
KPS score ≥80, *n* (%)	288 (90.9)	115 (74.7)	<0.001
ASA physical status, *n* (%)			<0.001
I-II	279 (88.0)	108 (70.1)	
III-IV	38 (12.0)	46 (29.9)	
Primary tumor site, *n* (%)			0.814
Esophageal/gastric	145 (45.7)	73 (47.4)	
Colorectal	172 (54.3)	81 (52.6)	
NRS2002 ≥3, *n* (%)	75 (23.7)	137 (89.0)	<0.001
GLIM malnutrition, *n* (%)	69 (21.8)	134 (87.0)	<0.001
Moderate malnutrition	41 (12.9)	54 (35.1)	<0.001
Severe malnutrition	20 (6.3)	65 (42.2)	<0.001
Albumin (g/L), mean ± SD	38.5 ± 4.2	34.1 ± 5.6	<0.001
Hemoglobin (g/L), mean ± SD	121.3 ± 18.5	109.8 ± 21.3	<0.001
CRP (mg/L), median (IQR)	8.5 (4.0, 15.2)	19.8 (9.5, 35.6)	<0.001

Data are presented as *n* (%), median (interquartile range), or mean ± standard deviation. *p*-values were calculated using the Chi-square test or Fisher’s exact test for categorical variables and the Mann–Whitney U test or independent samples *t*-test for continuous variables.

BMI, Body Mass Index; KPS, Karnofsky Performance Status; ASA, American Society of Anesthesiologists; NRS2002, Nutritional Risk Screening 2002; GLIM, Global Leadership Initiative on Malnutrition; CRP, C-reactive protein; IQR, Interquartile Range; SD, Standard Deviation.

Preoperative laboratory parameters also differed significantly between the groups. Patients who developed complications had significantly lower mean levels of serum albumin, hemoglobin, and hematocrit, and higher levels of C-reactive protein (CRP) compared to those in the complication-free group (all *p* < 0.05).

### 3.2 Comparison of hospitalization outcomes and readmission rates

Patients who experienced postoperative complications had substantially worse clinical and economic outcomes compared to those without complications. A detailed comparison is provided in Tables 2, 3. In summary, the complication group incurred significantly higher median hospitalization costs (RMB 75,500 vs. RMB 62,000; *p* < 0.001) and had a significantly longer median length of hospital stay (27.0 days vs. 22.0 days; *p* < 0.001). Furthermore, rates of ICU admission (8.44% vs. 0.63%; *p* < 0.001), 30-day unplanned readmission (10.39% vs. 1.89%; *p* < 0.001), and 60-day unplanned readmission (13.64% vs. 3.15%; *p* < 0.001) were all significantly higher in the complication group.

**Table 2:** Comparison of hospitalization costs and length of stay between patient groups.

Parameter	Without complications (*n* = 317)	With complications (*n* = 154)	Statistic (*Z*)	*p*-value
Hospitalization costs [RMB, median (IQR)]	62,000 [48,900–75,400]	75,500 [59,800–92,100]	−4.82	<0.001
Length of hospital stay [days, median (IQR)]	22.0 [18.0–28.0]	27.0 [21.0–35.0]	−3.710	<0.001

Data are presented as median (interquartile range). *p*-values were calculated using the Mann–Whitney U test.

RMB, Renminbi; IQR, Interquartile Range.

**Table 3:** Comparison of ICU admission and unplanned readmission rates.

Parameter (yes), *n* (%)	Without complications (*n* = 317)	With complications (*n* = 154)	*χ* ^2^	*p*-value
ICU admission rate	2 (0.63)	13 (8.44)	16.235	<0.001
30-day unplanned readmission rate	6 (1.89)	16 (10.39)	15.872	<0.001
60-day unplanned readmission rate	10 (3.15)	21 (13.64)	17.103	<0.001

Data are presented as *n* (%). *p*-values were calculated using the Chi-square test.

ICU, Intensive Care Unit.

### 3.3 Logistic regression analysis for postoperative complications

Univariable logistic regression analysis indicated that NRS2002-defined nutritional risk, GLIM-defined malnutrition, and GLIM malnutrition severity grades were significantly associated with an increased risk of postoperative complications. After adjusting for potential confounding factors (age, BMI, KPS score, ASA class, preoperative albumin, hemoglobin, and CRP) in the multivariable model, these associations remained statistically significant (Table 4).

**Table 4:** Logistic regression analysis of factors associated with postoperative complications.

Model	Variable	*β* Value	S.E.	Wald *χ*^2^	OR (95%CI)	*p*-value
Model 1 (unadjusted)						
NRS2002 (with risk vs. No risk)		2.03	0.24	73.27	7.62 (4.81–12.09)	<0.001
GLIM diagnosis (malnutrition vs. No)		1.73	0.23	58.52	5.65 (3.61–8.80)	<0.001
GLIM severity: moderate (vs. No)		1.56	0.27	32.49	4.79 (2.80–8.20)	<0.001
GLIM severity: severe (vs. No)		1.90	0.29	44.22	6.73 (3.84–11.81)	<0.001
Model 2 (adjusted^a^)						
NRS2002 (with risk vs. No risk)		2.03	0.27	57.46	7.58 (4.75–12.05)	<0.001
GLIM diagnosis (malnutrition vs. No)		1.73	0.26	44.89	5.62 (3.59–8.76)	<0.001
GLIM severity: moderate (vs. No)		1.56	0.30	27.56	4.78 (2.78–8.17)	<0.001
GLIM severity: severe (vs. No)		1.90	0.32	36.12	6.71 (3.82–11.78)	<0.001

a Model 2 adjusted for age, BMI, KPS score, ASA class, preoperative albumin, hemoglobin, and CRP. For the full model, Nagelkerke *R*^2^ was 0.385 and the Hosmer–Lemeshow test was non-significant (*p* = 0.21), indicating good model fit. No multicollinearity was detected (all VIF <2.5).

OR, Odds Ratio; CI, Confidence Interval; NRS2002, Nutritional Risk Screening 2002; GLIM, Global Leadership Initiative on Malnutrition; BMI, Body Mass Index; KPS, Karnofsky Performance Status; ASA, American Society of Anesthesiologists; CRP, C-reactive protein; VIF, Variance Inflation Factor.

Specifically, the odds of developing complications for patients with nutritional risk (NRS2002 ≥3) were 7.58 times the odds for those without nutritional risk (Adjusted OR 7.58, 95%CI: 4.75–12.05, *p* < 0.001). Similarly, the odds of complications for patients with GLIM-defined malnutrition were 5.62 times the odds for those without malnutrition (Adjusted OR 5.62, 95%CI: 3.59–8.76, *p* < 0.001). Compared to patients with no malnutrition, those with moderate malnutrition had an adjusted OR of 4.78 (95%CI: 2.78–8.17, *p* < 0.001), and those with severe malnutrition had an adjusted OR of 6.71 (95%CI: 3.82–11.78, *p* < 0.001) for postoperative complications.

**Figure 2:**
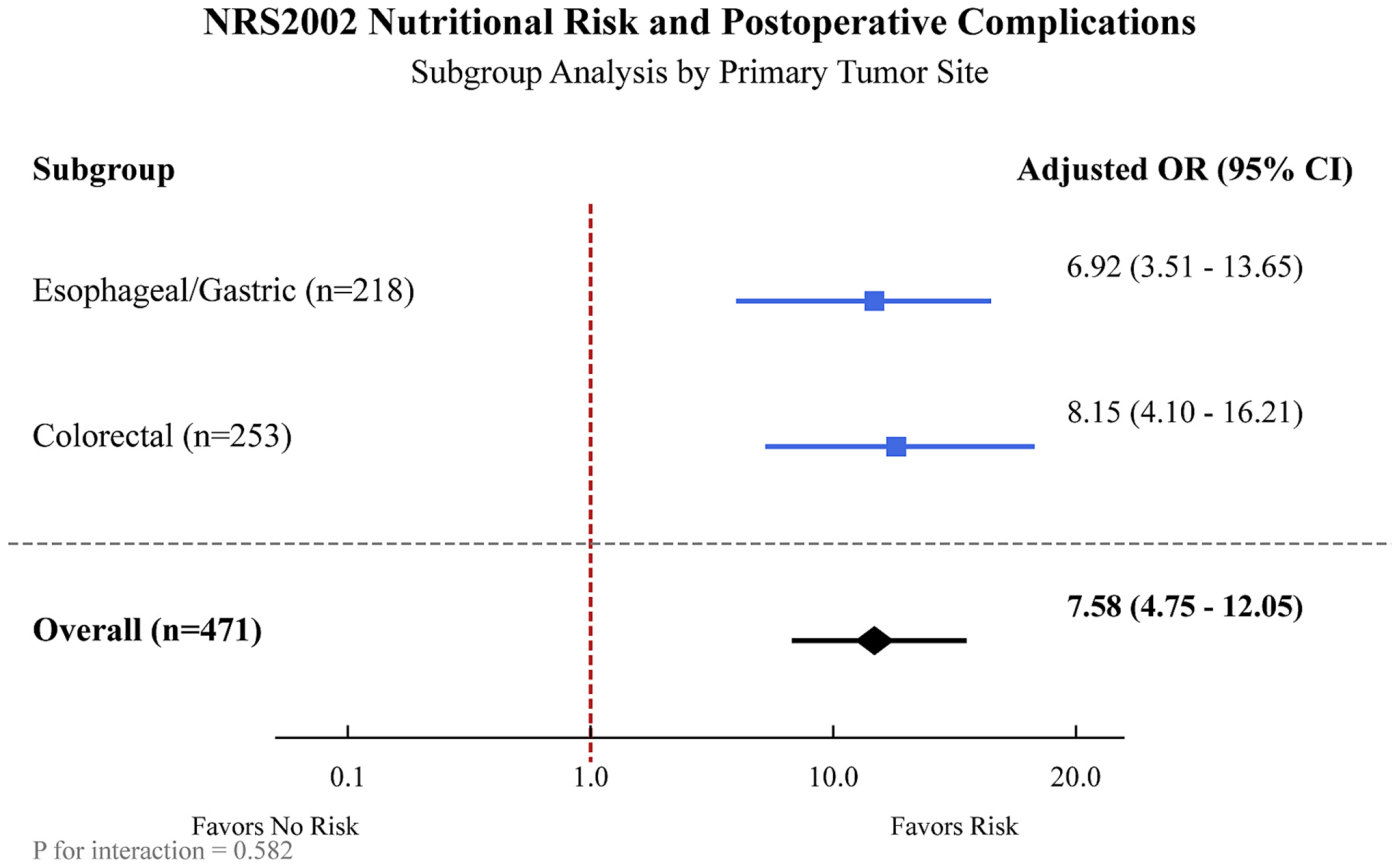
Subgroup analysis of the association between NRS2002 nutritional risk and postoperative complications, stratified by primary tumor site. The forest plot displays the adjusted odds ratios (ORs) and 95% confidence intervals (CIs) for postoperative complications associated with NRS2002 ≥ 3 (vs. <3) in patients with esophageal/gastric cancer and colorectal cancer. The overall OR represents the estimate for the entire cohort. Models were adjusted for age, BMI, KPS score, ASA class, preoperative albumin, hemoglobin, and CRP.

In a subgroup analysis stratified by primary tumor site, NRS2002 risk remained a significant predictor of postoperative complications in both patients with esophageal/gastric cancer (Adjusted OR 6.92, 95%CI: 3.51–13.65, *p* < 0.001) and those with colorectal cancer (Adjusted OR 8.15, 95%CI: 4.10–16.21, *p* < 0.001), with no significant interaction detected between tumor site and NRS2002 risk (*p* for interaction = 0.582) (Figure 2).

### 3.4 Logistic regression analysis for unplanned readmissions

Logistic regression analyses were conducted to evaluate the association between nutritional status (by NRS2002 and GLIM) and unplanned 30-day and 60-day readmissions. In both unadjusted and adjusted models (adjusted for age, KPS score, and primary tumor site), neither NRS2002-defined nutritional risk, GLIM-defined malnutrition, nor GLIM severity grading showed a statistically significant association with the risk of 30-day unplanned readmissions (Table 5) or 60-day unplanned readmissions (Table 6).

**Table 5:** Logistic regression analysis of factors associated with 30-day unplanned readmission.

Model	Variable	OR (95%CI)	*p*-value
Model 1 (unadjusted)			
NRS2002 (with risk vs. No risk)		1.70 (0.67–4.30)	0.261
GLIM diagnosis (malnutrition vs. No)		1.48 (0.58–3.69)	0.405
GLIM grading: moderate malnutrition (vs. No)		1.60 (0.51–4.90)	0.409
GLIM grading: severe malnutrition (vs. No)		1.80 (0.58–5.53)	0.305
Model 2 (adjusted^a^)			
NRS2002 (with risk vs. No risk)		1.65 (0.63–4.25)	0.290
GLIM diagnosis (malnutrition vs. No)		1.41 (0.54–3.67)	0.468
GLIM grading: moderate malnutrition (vs. No)		1.52 (0.47–4.88)	0.485
GLIM grading: severe malnutrition (vs. No)		1.71 (0.53–5.49)	0.364

a Model 2 adjusted for age, KPS score, and primary tumor site.

OR, Odds Ratio; CI, Confidence Interval; NRS2002, Nutritional Risk Screening 2002; GLIM, Global Leadership Initiative on Malnutrition; KPS, Karnofsky Performance Status.

**Table 6:** Logistic regression analysis of factors associated with 60-day unplanned readmission.

Model	Variable	OR (95%CI)	*p*-value
Model 1 (unadjusted)			
NRS2002 (with risk vs. No risk)		1.75 (0.72–4.40)	0.245
GLIM diagnosis (malnutrition vs. No)		1.52 (0.62–3.78)	0.380
GLIM grading: moderate malnutrition (vs. No)		1.63 (0.54–5.01)	0.390
GLIM grading: severe malnutrition (vs. No)		1.83 (0.62–5.62)	0.290
Model 2 (adjusted^a^)			
NRS2002 (with risk vs. No risk)		1.68 (0.68–4.15)	0.263
GLIM diagnosis (malnutrition vs. No)		1.45 (0.58–3.62)	0.421
GLIM grading: moderate malnutrition (vs. No)		1.55 (0.50–4.82)	0.443
GLIM grading: severe malnutrition (vs. No)		1.75 (0.58–5.28)	0.319

a Model 2 adjusted for age, KPS score, and primary tumor site.

OR, Odds Ratio; CI, Confidence Interval; NRS2002, Nutritional Risk Screening 2002; GLIM, Global Leadership Initiative on Malnutrition; KPS, Karnofsky Performance Status.

### 3.5 Predictive value of nutritional screening and assessment for postoperative complications

**Figure 3:**
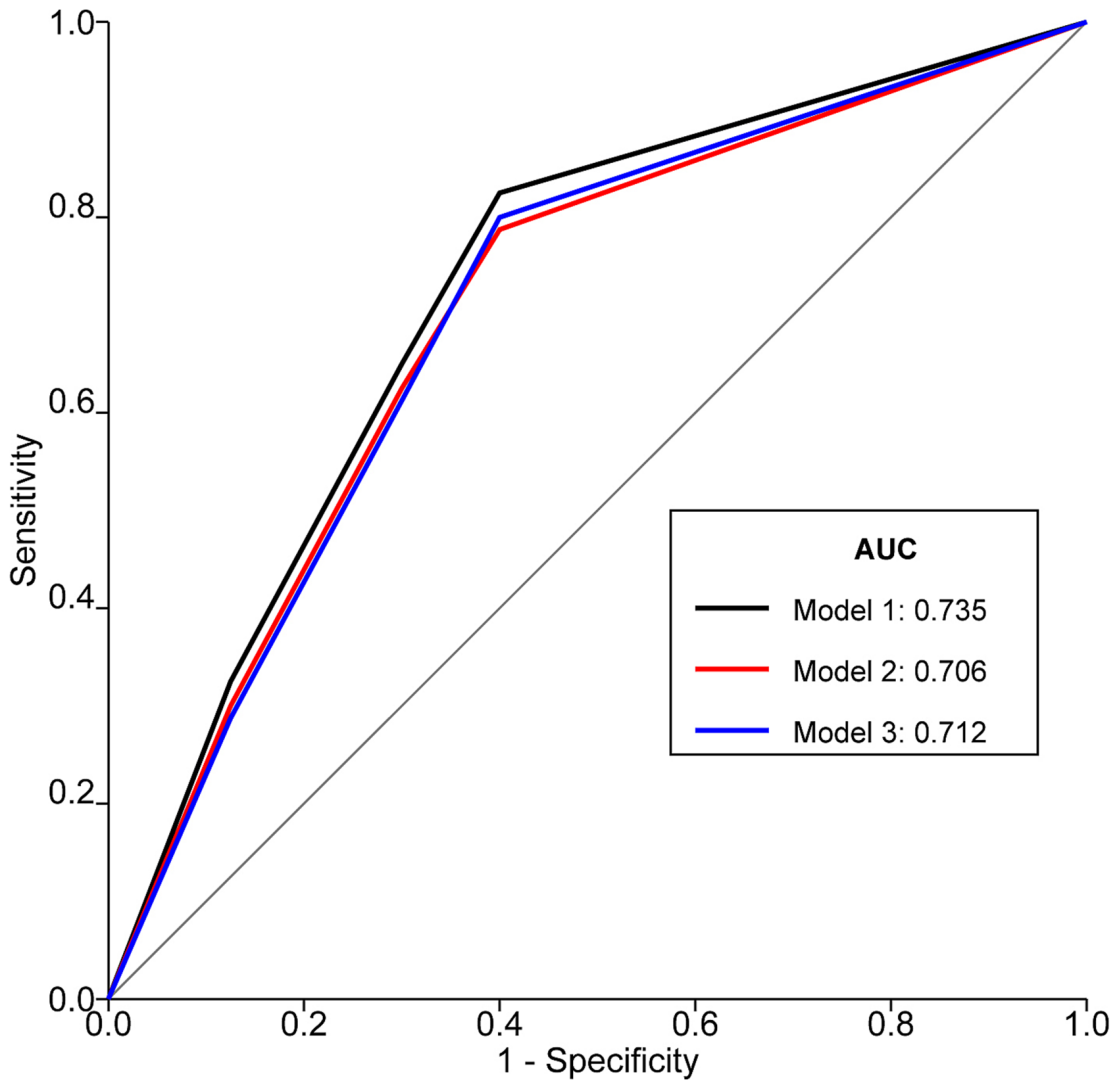
ROC curves of NRS2002, GLIM diagnosis, and GLIM severity grading for predicting postoperative complications in patients with Gastrointestinal Malignancies. Model 1 represents NRS2002 score, Model 2 represents dichotomous GLIM diagnosis (malnutrition vs. no malnutrition), and Model 3 represents GLIM severity grading (none, moderate, severe). AUCs were compared using DeLong’s test. Optimal cut-off for NRS2002 was ≥3 (Youden’s index = 0.469). ROC, Receiver Operating Characteristic; NRS2002, Nutritional Risk Screening 2002; GLIM, Global Leadership Initiative on Malnutrition; AUC, Area Under the Curve.

ROC curve analysis was employed to compare the predictive performance of NRS2002, GLIM diagnosis, and GLIM severity grading for postoperative complications (Figure 3). The AUC for NRS2002 score (Model 1) was 0.735 (95%CI: 0.691, 0.779; sensitivity: 76.6%, specificity: 70.3%; accuracy: 72.4%). The AUC for GLIM malnutrition diagnosis (Model 2) was 0.706 (95%CI: 0.660, 0.752; sensitivity: 70.8%, specificity: 70.3%; accuracy: 70.5%). The AUC for GLIM severity grading (Model 3) was 0.712 (95%CI: 0.663, 0.761; sensitivity: 70.2%, specificity: 70.3%; accuracy: 70.3%). All models were statistically significant (*p* < 0.001).

Comparison of AUCs revealed that Model 1 (NRS2002) had a significantly larger AUC than Model 2 (GLIM diagnosis) (*p* = 0.003). There were no statistically significant differences in AUC between Model 1 (NRS2002) and Model 3 (GLIM severity grading) (*p* = 0.215), nor between Model 2 (GLIM diagnosis) and Model 3 (GLIM severity grading) (*p* = 0.654).

## 4 Discussion

Malnutrition is a well-established adverse prognostic factor in patients with cancer, particularly those with GI malignancies, due to disease-related symptoms like obstruction, nausea, and bleeding, as well as tumor-induced metabolic derangements (21). Early identification and management of malnutrition are crucial. This study comprehensively evaluated the predictive utility of NRS2002 and GLIM criteria for postoperative outcomes in a cohort of 471 patients undergoing surgery for GI malignancies. Our findings confirm that both preoperative nutritional risk defined by NRS2002 and malnutrition defined by GLIM (including its severity grades) are significant independent predictors of postoperative complications.

Consistent with previous research (22), patients who developed postoperative complications in our cohort had significantly higher rates of NRS2002-defined nutritional risk and GLIM-defined malnutrition. These complications translated into tangible clinical burdens, including prolonged hospital stays, increased hospitalization costs, higher ICU admission rates, and increased rates of unplanned 30-day and 60-day readmissions, underscoring the clinical and economic impact of poor nutritional status.

The NRS2002 is a validated screening tool, but its performance can vary. A recent study involving elderly GI tumor patients reported that NRS2002-defined nutritional risk was not an independent predictor of complications after multivariable adjustment (23). In contrast, our study, which utilized the 2022 consensus for NRS2002 disease severity scoring (18) and included a broader adult age range, found that nutritional risk remained a strong independent predictor even after adjusting for multiple confounders. This discrepancy might be attributable to differences in patient populations, methodological approaches to NRS2002 scoring, or the range of confounders considered. The updated consensus on disease severity scoring may enhance the sensitivity of NRS2002, potentially capturing at-risk patients more effectively, especially in complex cases with multiple comorbidities, which are increasingly common, as recently highlighted in surgical oncology research (24).

The GLIM criteria represent a global effort to standardize malnutrition diagnosis (13). A recent study confirmed that GLIM-defined malnutrition is a robust predictor of adverse postoperative outcomes across various surgical disciplines, as demonstrated by Murnane et al. (25), who found that GLIM criteria identified a significantly higher incidence of malnutrition (72.2%) compared to ICD-10 criteria (40.7%, *p* < 0.001) in patients undergoing oesophagogastric cancer surgery, and this was strongly associated with increased risks of pulmonary complications such as pneumonia [odds ratio (OR): 5.17, *p* = 0.034] and pleural effusions (26.9% vs. 6.7%, *p* = 0.010), while multivariate analyses confirmed GLIM-defined malnutrition as an independent predictor of these outcomes after adjusting for confounding variables, highlighting its superior clinical utility in risk stratification and postoperative prognosis assessment. Our findings align with this, demonstrating that GLIM-defined malnutrition, and its severity, are independent risk factors for postoperative complications in GI cancer surgery patients. This is clinically intuitive, as the phenotypic criteria of GLIM (weight loss, low BMI, reduced muscle mass) are core components of cancer cachexia, which is known to impair recovery (4, 26). The etiologic criterion of inflammation, inherent in malignancy, further compounds this risk. Our analysis of GLIM severity showed a graded relationship, with severe malnutrition conferring a higher risk than moderate malnutrition, emphasizing the importance of not only diagnosing but also staging malnutrition. This graded risk is consistent with findings from other surgical cohorts, where severe GLIM-defined malnutrition has been linked to higher rates of major complications (26.9% vs. 6.7% for pneumonia), anastomotic leakage, and mortality (HR: 2.51, *p* = 0.014) compared to moderate malnutrition (25).

In terms of predictive accuracy for postoperative complications, NRS2002 demonstrated a slightly, but statistically significantly, better AUC than the dichotomous GLIM diagnosis (malnourished vs. not malnourished). This marginal superiority may be attributed to the inclusion of age (for patients ≥70 years) and a graded disease severity score in the NRS2002 calculation, which provides a more nuanced risk assessment compared to the binary etiologic criteria of GLIM (27). However, when GLIM malnutrition was graded by severity, its AUC was comparable to that of NRS2002. This suggests that while NRS2002 may have a marginal edge in overall risk stratification for complications in this population, a graded GLIM assessment offers similar predictive utility. Both tools provide valuable prognostic information. The choice of tool may depend on clinical workflow, available resources for detailed GLIM assessment (e.g., muscle mass measurement), and specific research or clinical objectives (28, 29).

Interestingly, neither NRS2002 nor GLIM criteria were found to be significantly associated with unplanned 30-day or 60-day readmissions in our adjusted analyses. This finding contrasts with some studies that have linked poor nutritional status to higher readmission rates (30). The primary reasons for readmission in our cohort included GI obstruction, anastomotic issues, and stoma-related complications, which, while potentially influenced by nutritional status, are also heavily dependent on surgical technique and acute postoperative events. It is also plausible that our exclusion of patients returning for planned adjuvant therapies (radiotherapy/chemotherapy) might have influenced these results, as such patients could represent a subgroup where nutritional impact on readmission is more pronounced but are not “unplanned” readmissions in the same sense. Furthermore, a low event rate for readmissions may have limited the statistical power to detect a significant association. Further research specifically targeting risk factors for unplanned readmissions in this population is warranted.

This study has several strengths, including its prospective design, a relatively large sample size for a single-center study, the use of standardized nutritional assessment tools including the latest GLIM criteria and updated NRS2002 guidance, and comprehensive data collection on outcomes. However, limitations must be acknowledged. First, being a single-center study, the findings may not be generalizable to all populations or healthcare settings. Second, while we adjusted for numerous confounders, residual confounding cannot be entirely excluded. Third, the assessment of muscle mass was heterogeneous; reliance on anthropometric proxies for a majority of patients, while practical, is less precise than standardized imaging techniques like CT. This could potentially underestimate the prevalence of low muscle mass and slightly blunt the predictive power of the GLIM criteria. Fourth, this study was designed to compare the predictive performance of NRS2002 and GLIM when applied independently to all patients. While a sequential approach (screening with NRS2002, followed by GLIM diagnosis for those at risk) is a recommended clinical workflow, our parallel application was intended to evaluate each tool’s inherent prognostic value in this specific research context. Finally, we conducted a sensitivity analysis by excluding the 23 ungraded malnutrition patients from the dichotomous GLIM analysis, which yielded similar results (Adjusted OR 5.49, 95% CI: 3.48–8.66, *p* < 0.001), confirming the robustness of our findings. It is important to clarify that these 23 patients had sufficient data to meet the GLIM diagnostic criteria, thus not meeting the study’s exclusion criterion for ‘incomplete critical data.’ The ambiguity arose only in the final severity grading step due to borderline or conflicting phenotypic indicators, which is a known challenge in complex clinical cases. Lastly, the early discharge of 7 patients within 48 h post-surgery meant their complication status could not be assessed, representing a small loss to follow-up for the primary outcome. Future multi-center studies with larger cohorts and standardized, advanced body composition analysis are needed to further validate these findings and explore the nuances of nutritional impact on diverse postoperative outcomes.

## 5 Conclusion

Preoperative nutritional risk identified by NRS2002 and malnutrition diagnosed by GLIM criteria (including its severity grades) are significant, independent risk factors for the development of postoperative complications in patients undergoing surgical resection for gastrointestinal malignancies. The NRS2002 score demonstrated a slightly superior predictive ability for overall postoperative complications compared to the dichotomous GLIM malnutrition diagnosis, although GLIM severity grading showed comparable predictive performance to NRS2002. Neither tool was significantly associated with unplanned 30-day or 60-day readmissions in this cohort. Routine preoperative nutritional screening and assessment using tools like NRS2002 and GLIM are essential for identifying high-risk patients who may benefit from targeted perioperative nutritional interventions to mitigate complication risks and improve surgical outcomes.

## Data Availability

The original contributions presented in the study are included in the article/supplementary material, further inquiries can be directed to the corresponding author.

## References

[1] Kocis J Wendsche P Muzík V Bilik A Veselý R Cernohousová I. Minimally invasive thoracoscopic transdiaphragmatic approach to thoracolumbar junction fractures. Acta Chir Orthop Traumatol Cechoslov. (2009) 76:232–8. doi: 10.55095/achot2009/042, PMID: 19595286

[2] Li X Xiao X Wu Z Li A Wang W Lin R. Global, regional, and national burden of early-onset colorectal cancer and projection to 2050: an analysis based on the global burden of disease study 2021. Public Health. (2025) 238:245–53. doi: 10.1016/j.puhe.2024.12.011, PMID: 39700867

[3] Arends J Bachmann P Baracos V Barthelemy N Bertz H Bozzetti F. ESPEN guidelines on nutrition in cancer patients. Clin Nutr. (2017) 36:11–48. doi: 10.1016/j.clnu.2016.07.015, PMID: 27637832

[4] Weimann A Braga M Carli F Higashiguchi T Hübner M Klek S. ESPEN practical guideline: clinical nutrition in surgery. Clin Nutr. (2021) 40:4745–61. doi: 10.1016/j.clnu.2021.03.031, PMID: 34242915

[5] Correia MI Waitzberg DL. The impact of malnutrition on morbidity, mortality, length of hospital stay and costs evaluated through a multivariate model analysis. Clin Nutr. (2003) 22:235–9. doi: 10.1016/S0261-5614(02)00215-7, PMID: 12765661

[6] Schiesser M Müller S Kirchhoff P Breitenstein S Schäfer M Clavien PA. Assessment of a novel screening score for nutritional risk in predicting complications in gastro-intestinal surgery. Clin Nutr. (2008) 27:565–70. doi: 10.1016/j.clnu.2008.01.010, PMID: 18342995

[7] Cederholm T Barazzoni R Austin P Ballmer P Biolo G Bischoff SC. ESPEN guidelines on definitions and terminology of clinical nutrition. Clin Nutr. (2017) 36:49–64. doi: 10.1016/j.clnu.2016.09.004, PMID: 27642056

[8] Barazzoni R Jensen GL Correia M Correia MITD Gonzalez MC Higashiguchi T. Guidance for assessment of the muscle mass phenotypic criterion for the global leadership initiative on malnutrition (GLIM) diagnosis of malnutrition. Clin Nutr. (2022) 41:1425–33. doi: 10.1016/j.clnu.2022.02.001, PMID: 35450768

[9] Chinese Society for Parenteral and Enteral Nutrition. Guidelines on nutritional support in patients with tumor. Zhonghua Wai Ke Za Zhi. (2017) 55:801–29. doi: 10.3760/cma.j.issn.0529-5815.2017.11.001, PMID: 29136728

[10] Kondrup J Rasmussen HH Hamberg O Stanga Z. Nutritional Risk Screening (NRS 2002): a new method based on an analysis of controlled clinical trials. Clin Nutr. (2003) 22:321–36. doi: 10.1016/s0261-5614(02)00214-5, PMID: 12765673

[11] Skipper A Ferguson M Thompson K Castellanos VH Porcari J. Nutrition screening tools: an analysis of the evidence. JPEN J Parenter Enteral Nutr. (2012) 36:292–8. doi: 10.1177/0148607111414023, PMID: 22045723

[12] Cederholm T Jensen GL Correia M Correia MITD Gonzalez MC Fukushima R. Glim criteria for the diagnosis of malnutrition—a consensus report from the global clinical nutrition community. Clin Nutr. (2019) 38:1–9. doi: 10.1016/j.clnu.2018.08.002 30181091

[13] Cederholm T Jensen GL Correia M Correia MITD Gonzalez MC Fukushima R. Glim criteria for the diagnosis of malnutrition—a consensus report from the global clinical nutrition community. J Cachexia Sarcopenia Muscle. (2019) 10:207–17. doi: 10.1002/jcsm.12383, PMID: 30920778 PMC6438340

[14] Pumtako C Dolan RD McMillan DC. Prevalence and prognostic value of global leadership initiative on malnutrition (GLIM) phenotypic cachexia criteria in cancer patients: a systematic review and meta-analysis. Clin Nutr ESPEN. (2025) 67:387–97. doi: 10.1016/j.clnesp.2025.03.044, PMID: 40147760

[15] Zhang KP Zhang X Zhang Q Ruan GT Song MM Xie HL. Association between the lymphocyte-to-C-reactive protein ratio and survival outcomes in Cancer patients with GLIM-defined malnutrition: a multicenter study. J Nutr Health Aging. (2022) 26:847–55. doi: 10.1007/s12603-022-1835-3, PMID: 36156676 PMC12280780

[16] Takimoto M Yasui-Yamada S Nasu N Kagiya N Aotani N Kurokawa Y. Development and validation of cutoff value for reduced muscle mass for GLIM criteria in patients with gastrointestinal and hepatobiliary-pancreatic cancers. Nutrients. (2022) 14:943. doi: 10.3390/nu14050943, PMID: 35267918 PMC8912591

[17] Qin L Tian Q Zhu W Wu B. The validity of the GLIM criteria for malnutrition in hospitalized patients with gastric Cancer. Nutr Cancer. (2021) 73:2732–9. doi: 10.1080/01635581.2020.1856894, PMID: 33305620

[18] Zhejiang Medical Doctor Association Committee of Nutrition Physicians ZCNCZMABoP N E. Expert consensus on Nutritional Risk Screening and disease severity scoring. Zhejiang Med J. (2022) 44:1351–61.

[19] Dindo D Demartines N Clavien PA. Classification of surgical complications: a new proposal with evaluation in a cohort of 6336 patients and results of a survey. Ann Surg. (2004) 240:205–13. doi: 10.1097/01.sla.0000133083.54934.ae, PMID: 15273542 PMC1360123

[20] Li ZY Wu ZQ Ji JF. Expert consensus on the diagnostic and registration criteria for postoperative complications of gastrointestinal tumor surgery in China (2018 edition). Chin J Pract Surg. (2018) 38:7.

[21] Fearon K Strasser F Anker SD Bosaeus I Bruera E Fainsinger RL. Definition and classification of cancer cachexia: an international consensus. Lancet Oncol. (2011) 12:489–95. doi: 10.1016/S1470-2045(10)70218-7, PMID: 21296615

[22] Xi Z Jia-Xin H Xi W Meng T Jin-Feng L Wei W. Association between GLIM-diagnosed malnutrition and quality of life in older patients with cancer. J Nutr Health Aging. (2024) 28:100274. doi: 10.1016/j.jnha.2024.100274, PMID: 38810512 PMC12275700

[23] Li YF Nie RC Wu T Li SM Chen S Wang W. Prognostic value of the Nutritional Risk Screening 2002 scale in metastatic gastric Cancer: a large-scale cohort study. J Cancer. (2019) 10:112–9. doi: 10.7150/jca.27729, PMID: 30662531 PMC6329866

[24] Limbu Y Raut S Pudasaini P Regmee S Ghimire R Maharjan DK. Correlation of the Nutritional Risk Screening 2002 score with post-operative complications in gastrointestinal and Hepatopancreatobiliary Oncosurgeries. Cureus. (2024) 16:e58514. doi: 10.7759/cureus.58514, PMID: 38957834 PMC11218454

[25] Murnane LC Forsyth AK Koukounaras J Shaw K King S Brown WA. Malnutrition defined by GLIM criteria identifies a higher incidence of malnutrition and is associated with pulmonary complications after oesophagogastric cancer surgery, compared to ICD-10-defined malnutrition. J Surg Oncol. (2023) 128:769–80. doi: 10.1002/jso.27366, PMID: 37291908

[26] Martin L Senesse P Gioulbasanis I Antoun S Bozzetti F Deans C. Diagnostic criteria for the classification of cancer-associated weight loss. J Clin Oncol Off J Am Soc Clin Oncol. (2015) 33:90–9. doi: 10.1200/JCO.2014.56.1894, PMID: 25422490

[27] Xie B Sun Y Sun J Deng T Jin B Gao J. Applicability of five nutritional screening tools in Chinese patients undergoing colorectal cancer surgery: a cross-sectional study. BMJ Open. (2022) 12:e057765. doi: 10.1136/bmjopen-2021-057765, PMID: 35623749 PMC9150165

[28] Trollebø MA Skeie E Revheim I Stangeland H Erstein MAH Grønning MK. Comparison of Nutritional Risk Screening with NRS2002 and the GLIM diagnostic criteria for malnutrition in hospitalized patients. Sci Rep. (2022) 12:19743. doi: 10.1038/s41598-022-23878-3, PMID: 36396666 PMC9672100

[29] Mareschal J Achamrah N Norman K Genton L. Clinical value of muscle mass assessment in clinical conditions associated with malnutrition. J Clin Med. (2019) 8:1040. doi: 10.3390/jcm8071040, PMID: 31319519 PMC6678556

[30] Read JA Choy ST Beale PJ Clarke SJ. Evaluation of nutritional and inflammatory status of advanced colorectal cancer patients and its correlation with survival. Nutr Cancer. (2006) 55:78–85. doi: 10.1207/s15327914nc5501_10, PMID: 16965244

